# Comprehensive elucidation of volatile, phenolic, and organic acid profiles in Turkish mallow (*Malva sylvestris* L.) petal herbal tea: impact of diverse brewing conditions

**DOI:** 10.1002/jsfa.70881

**Published:** 2026-07-06

**Authors:** Armin Amanpour, Ali Tekin, Ali Adnan Hayaloglu

**Affiliations:** ^1^ Department of Gastronomy and Culinary Arts, Faculty of Fine Arts Design and Architecture Istanbul Medipol University Istanbul Türkiye; ^2^ Department of Food Engineering Inonu University Malatya Türkiye; ^3^ Department of Food Processing, Vocational School of Keban Fırat University Elazığ Türkiye

**Keywords:** bioactive compounds, functional foods, herbal tea, infusion conditions, *Malva sylvestris*, volatile and phenolic compounds

## Abstract

**BACKGROUND:**

Mallow (*Malva sylvestris* L.) petals are traditionally consumed as herbal tea across the Mediterranean for their soothing and anti‐inflammatory properties; however, scientific characterization of this consumption‐relevant matrix under realistic brewing conditions remains scarce. This study comprehensively elucidates the impact of three household infusion protocols, short hot brewing (4 min at 98 °C), extended hot brewing (16 min at 98 °C), and cold brewing (24 h at 25 °C), on the volatile, phenolic, and organic acid profiles, antioxidant capacity, and sensory attributes of Turkish endemic *M. sylvestris* petal tea.

**RESULTS:**

Using liquid chromatography–tandem mass spectrometry (LC–MS/MS) and gas chromatography–mass spectrometry (GC–MS), coupled with univariate (one‐way analysis of variance (ANOVA) with Duncan's test, effect size) and multivariate (principal component analysis (PCA), Spearman rank correlation) statistics, distinct phytochemical fingerprints were identified for each method. Cold infusion yielded the highest total phenolic and total flavonoid contents and superior ABTS activity, while short hot infusion best preserved esters and terpenes, correlating strongly with high hedonic scores for ‘Aroma’ and ‘Overall Liking.’ Extended hot infusion promoted lipid oxidation products (e.g., nonanal) and partial degradation of thermolabile antioxidants. PCA revealed clear separation of samples by infusion type, and Spearman correlations linked specific volatiles (e.g., ethyl palmitate, nonanoic acid) and phenolics (e.g., gallic acid, protocatechuic acid) to sensory and functional outcomes.

**CONCLUSION:**

Changes in brewing time and temperature significantly affect the bioactive and sensory quality of teas. Short hot infusion offers superior sensory scores, ideal for daily consumption. Cold brewing method yields higher phenolics but lower sensory appeal, preferable for health. Extended hot infusion compromises both quality and function. These findings guide balance between health benefits and sensory quality. © 2026 The Author(s). *Journal of the Science of Food and Agriculture* published by John Wiley & Sons Ltd on behalf of Society of Chemical Industry.

## INTRODUCTION

Mallow (*Malva sylvestris* L., Malvaceae) is a biennial‐perennial medicinal plant native to Europe, North Africa, Southwest Asia, and Türkiye. Among the approximately 40 *Malva* species, *M. sylvestris* is notable for its extensive ethnomedicinal use and cultural relevance across the Mediterranean basin. Traditionally, its violet‐purple petals, marked by dark venation, are predominantly consumed as herbal tea to relieve gastrointestinal discomfort, respiratory ailments, and inflammatory conditions.^1^, ^2^ Despite this widespread traditional use of petals, most phytochemical studies on *M. sylvestris* have focused on leaves, stems, or whole aerial parts, likely due to their greater biomass yield and ease of collection during full‐plant harvests.^3^, ^4^ Consequently, the petal, the actual matrix consumed in daily practice, remains underexplored in terms of its infusion‐dependent bioactive and sensory dynamics.

Herbal teas, which enable the aqueous extraction of bioactive compounds during infusion, are a common way that medicinal plants are consumed worldwide. *Malva sylvestris* petals are usually dried (e.g., sun or shade drying) prior to infusion to preserve their phytochemical and aromatic integrity. The release, transformation, and stability of volatile compounds, which are essential for the sensory quality and consumer acceptance of herbal teas, are greatly influenced by drying and post‐infusion conditions.^5^ For example, drying alters volatile profile of *Hibiscus sabdariffa* L., resulting in the production and breakdown of aroma compounds.^5^ The sensory quality of herbal tea is strongly affected by volatile compounds, which directly influence freshness, flavor, and aroma. Their various chemical classes (such as acids, alcohols, aldehydes, esters, ketones, terpenes, lactones, phenols, and furans) contribute to unique olfactory characteristics, including fruity, floral, green, fatty, and spicy notes.^6^, ^7^ Although *M. sylvestris* petal tea is popular for its pleasant aroma, there is limited information in the literature about its volatile profile and essential odorants, particularly regarding to changes caused by infusion.^8^ Hexadecanoic acid, pentacosane, and 6,10,14‐trimethyl‐2‐pentadecanone have been identified as important constituents in major studies on the volatile compounds of *M. sylvestris* which have primarily examined essential oils from dried petals.^8^ However, this approach does not fully clarify the aroma profile directly related to aqueous infusions, which involve different extraction dynamics for water‐soluble aroma compounds.

Petals of *M. sylvestris* are an important source of organic and phenolic acids as well as volatile compounds. Liquid chromatography–tandem mass spectrometry (LC–MS/MS) analyses have identified key phenolics such as rutin, rosmarinic, caffeic, chlorogenic, and tannic acids, although citric, fumaric, oxalic, and malic acids usually predominate the organic acid profile.^9^, ^10^ These compounds enhance the plant's antioxidant potential and significantly influence basic physicochemical properties such as color and pH, which are important factors in determining overall quality and consumer perception.

Although *M. sylvestris* has been thoroughly studied in various extract forms including leaves, stems, and seeds little is known about its most widely consumed form for human consumption: herbal tea made from petals. By evaluating *M. sylvestris* petal tea under conventional infusion conditions, this study aims to address this research gap and provide a new perspective on the tea's actual bioactive and sensory characteristics as traditionally prepared and consumed. This approach enables a direct understanding of how different preparation techniques affect the chemical composition and functional properties of the final product.

Cold brewing for 24 h at room temperature (25 °C) (24H), short hot brewing for 4 min at 98 °C (4M), and extended hot brewing for 16 min at 98 °C (16M) were selected as representative protocols reflecting common household practices and established methodologies for petal herbal teas, capturing kinetic, equilibrium, and ambient infusion regimes reported in the literature.^6^, ^11^, ^12^, ^13^ The 24H condition, while consumed in some traditional practices (e.g., overnight infusion), was also included as an extreme infusion endpoint to characterize time‐dependent release kinetics of bioactive compounds, consistent with established methodologies in herbal tea research.^6^, ^11^, ^12^, ^14^, ^15^ This study investigates how brewing conditions influence the phenolic, volatile, and organic acid profiles, antioxidant capacity (using both 2,2‐diphenyl‐1‐picrylhydrazyl (DPPH) and 2,2′‐azino‐bis (3‐ethylbenzothiazoline‐6‐sulfonic acid) (ABTS) methods), and sensory attributes of herbal tea prepared from petals of the Turkish endemic *M. sylvestris*. To the authors' knowledge, this is the first integrated assessment of *M. sylvestris* petal tea under consumption‐relevant brewing scenarios, combining comprehensive phytochemical profiling with multivariate statistical interpretation to bridge traditional use and scientific characterization, providing essential information about the bioactive and sensory characteristics of the tea.

## MATERIALS AND METHODS

### Plant material and chemicals

Fresh petals of mallow (*M. sylvestris* L.) were collected from Urla, Izmir, in western Türkiye during the 2025 season. To minimize confounding variables and isolate the effect of brewing parameters, petals were sourced from this single, well‐documented location. The petals were immediately shade‐dried under ambient conditions (25 ± 2 °C, protected from direct sunlight) for approximately 7 days until constant weight was achieved, following established protocols for preserving volatile integrity in floral herbal materials.^11^, ^12^ The dried petals were then sorted to remove impurities, packed in small polyethylene bags, and stored at 4 °C until further use. Distilled water was prepared using a Milli‐Q system (Millipore, Billerica, MA, USA). Distilled dichloromethane, sodium sulfate, methanol (high‐performance liquid chromatography (HPLC) grade), formic acid, sodium carbonate, and hydrochloric acid, all obtained from Merck (Darmstadt, Germany), were used for the analyses. Standard volatile and phenolic compounds were supplied by Sigma‐Aldrich (St Louis, MO, USA).

### Herbal tea sample preparation

Three different herbal tea varieties of mallow (*M. sylvestris* L.), designated as 4M, 16M, and 24H were prepared from previously dried petals. These variations represent three distinct brewing techniques: cold brewing (24 h at room temperature (25 °C)), extended hot brewing (16 min at 98 °C), and short hot brewing (4 min at 98 °C). The hot brewing temperature of 98 °C, achieved by allowing freshly boiled water to cool briefly before brewing, was selected to ensure controlled near‐boiling extraction while avoiding full boiling. This temperature aligns with commonly used conditions in both standardized procedures, which enhance antioxidant and phenolic extraction while avoiding excessive thermal degradation of heat‐sensitive compounds,^16^ and typical household practices validated by consumer acceptance studies.^17^ Additionally, this temperature has been widely employed in previous infusion studies, including white,^16^ black,^17^ and herbal^6^ teas. The temperature was carefully maintained using a thermostatically controlled water bath throughout the infusion process to avoid full boiling. Briefly, 2 g of dried mallow petals were steeped in 150 mL of distilled water at 98 °C for 4 min for the 4M sample and 16 min for the 16M sample. For the 24H sample, 2 g of mallow petals were immersed in 150 mL of pre‐cooled distilled water at 25 °C for 24 h. The 4M and 16M hot tea infusions were brewed and then immediately cooled to room temperature.

### Physicochemical analysis

The oven‐drying method was used to determine the dry matter content of the dried petals.^18^ A digital pH meter (Seven Compact TMS220; Mettler Toledo, Schwerzenbach, Switzerland) was used to measure the pH of the herbal tea samples. Each herbal tea sample was evaluated with a colorimeter (Chroma Meter CR‐5; Konica Minolta, Tokyo, Japan) to determine color parameters such as L* (lightness), a* (red/greenness), and b* (yellow/blueness) values. Measurements were made in triplicate.^19^

### Extraction of volatile compounds

The solvent‐assisted flavor evaporation (SAFE) unit (Glasbläserei Bahr, Manching, Germany) equipped with a DCP 3000 Vacuubrand GmbH (Wertheim, Germany) and operating under high vacuum (10–3 Pa) was used to extract volatile compounds from all herbal tea samples. This extraction technique was originally developed by Engel *et al*. for isolating volatile compounds from complex food matrices.^20^ It has since been successfully applied to a variety of plant‐derived food and beverage matrices, including saffron (*Crocus sativus* L.),^21^ olive oils,^22^ and tea (*Camellia sinensis*),^23^ confirming its suitability and efficiency for such applications. The technique was subsequently adapted from our earlier studies.^21^, ^22^ For each extraction, a 40 mL aliquot of the mallow herbal tea sample, 100 mL of dichloromethane as a solvent, and 10 μL of 2‐methyl‐3‐heptanone as an internal standard were combined. 2‐Methyl‐3‐heptanone was selected as the internal standard because it does not naturally occur in the tea matrices analyzed, exhibits stable chromatographic behavior under the gas chromatography–mass spectrometry (GC–MS) conditions used, and provides a consistent, well‐resolved signal that enables accurate normalization of volatile compound peak areas. This compound is widely employed as an internal standard in volatile analysis of various food matrices.^24^, ^25^ Dichloromethane was chosen as the extraction solvent for its effectiveness in isolating volatile compounds.^21^, ^22^ The mixture was stirred with a magnetic stirrer at moderate speed (approximately 700 rpm) for 30 min in a nitrogen atmosphere, then centrifuged at 5500 rpm (approximately 3500 × *g*) for 15 min at 4 °C.

The upper transfer head unit of the SAFE system was gradually filled with the resulting organic phase. As the sample contents fell into a round‐bottom flask partially immersed in a water bath at 40 °C, the volatile compounds separated. After passing through the separation head and into a receiving tube, the separated volatile components froze and condensed due to the sharp drop in temperature. When the separation procedure was complete, the receiving tube was removed and thawed for 30 min at room temperature.^20^ The thawed organic extract was then dehydrated with anhydrous sodium sulfate. The extract was then concentrated to a final volume of 5 mL using a Kuderna–Danish concentrator (Sigma‐Aldrich) with a Snyder column at 40 °C (Supelco, St Quentin, France). Further concentration to 200 μL was achieved under a gentle stream of purified nitrogen. Three separate extractions were performed for each herbal tea sample. Prior to chromatographic analysis, the concentrated extracts were stored in 2 mL glass vials at −20 °C.

### GC–MS analysis of volatile compounds

A Shimadzu GC‐2010 gas chromatograph and QP‐2010 mass spectrometer (Shimadzu Corporation, Kyoto, Japan) were used to identify and quantify volatile compounds. Chromatographic separation was performed on a DB‐Wax column (DB‐5MS, 60 m × 0.25 mm inner diameter × 0.25 μm film thickness; J&W Scientific, Folsom, CA, USA). The GC oven temperature program began with an isothermal hold at 40 °C for 2 min, serving as the desorption time. The temperature then increased to 70 °C at a rate of 5 °C min^−1^ and held for 1 min. A final heating gradient of 10 °C min^−1^ was applied to reach 240 °C, resulting in a total run time of 30 min.

With a threshold of 1000 and a sampling rate of 1.11 scans s^−1^, the MS system was run in scan mode, recording ions in the mass range of 33 to 450 atomic mass units (amu). By comparing the mass spectra of the volatile compounds with entries in the NIST/EPA/NIH 02 (http://www.nist.gov) and Wiley (7th edition, 2005) spectral libraries, to identify and confirm the compounds. Calculating retention indices (RIs) and comparing them to authentic chemical standards was necessary for further confirmation. By co‐injecting an n‐alkane series (C8–C20) under the same chromatographic conditions, the RIs for each compound were calculated. In addition, a panel of 30 reference standard compounds (Sigma Chemical Co., St Louis, MO, USA) was used to definitively verify the identity of volatile compounds present in the samples.

### Reverse‐phased HPLC analysis of phenolic compounds and antioxidant capacity

Prior to HPLC analysis and antioxidant activity, phenolic extracts directly from herbal tea infusions were filtered through a 0.45 μm pore size polyvinylidene fluoride (PVDF) membrane filter.

#### Determination of phenolic compounds

A Shimadzu LC 20AD Prominence HPLC system (Shimadzu Corporation) was used to determine the phenolic content of the herbal tea infusions. The system was equipped with DGU‐20A5 degasser units, a CTO‐20A column heater, a SIL‐20A HT autosampler, and an SPD‐M20A photodiode array (PDA) detector. A C18 ODS‐3 column (250 mm × 4.6 mm internal diameter, 5 μm particle size; GL Sciences, Kyoto, Japan) was used for chromatographic separation. Following the methodology developed in our previously published work, the precise chromatographic parameters for the separation and detection of phenolic compounds were established.^26^ Phenolic compounds were identified by comparing their retention times and ultraviolet (UV)‐visible absorption spectra with those of authentic external standards (Sigma‐Aldrich; ≥ 99% purity) analyzed under identical chromatographic conditions. For compounds lacking commercially available reference standards (e.g., certain glycosylated derivatives), tentative identification was achieved using high‐resolution mass spectrometry (HRMS) data in conjunction with retention time comparison against structurally analogous standards and previously reported literature values.^3^, ^4^ Quantification was performed using calibration curves of the closest available aglycone or acid standard when the exact conjugate was unavailable. Each analysis was performed in triplicate.

#### Evaluation of antioxidant capacity

Both DPPH and ABTS radical scavenging assays were used to evaluate the antioxidant potential of the prepared herbal tea samples. For the ABTS assay, the antioxidant capacity was determined based on the decolorization of the ABTS radical cation, following a detailed analytical method previously established by our research group.^26^ Absorbance measurements were taken at 734 nm, and the results were compared to several Trolox standard solutions. Results were expressed as milligrams of Trolox equivalent per kilogram of herbal tea. The DPPH radical scavenging activity was measured at 517 nm using an analytical technique that was thoroughly described in our previous study.^26^ The results from the DPPH assay were also expressed as milligrams of Trolox equivalent per kilogram of herbal tea.

### Reversed‐phase HPLC analysis of organic acids

A reversed‐phase HPLC system (Shimadzu LC 20AD; Shimadzu Corporation) was used to determine the organic acid composition of the herbal tea samples. An aliquot of the mallow herbal tea sample was carefully filtered through a PVDF membrane filter with a pore size of 0.45 μm for this analysis. To separate the organic acids, 20 μL of the filtered sample was injected onto an HPLC column (Hi‐Plex 7.7 mm × 300 mm, 8 μm; Agilent Technologies, Santa Clara, CA, USA). A PDA detector set at 210 nm was used to identify these acids. Organic acids were identified by co‐injection with pure analytical standards (Sigma‐Aldrich; ≥ 99% purity) under identical chromatographic conditions. Peak identity was confirmed by matching both retention time and UV absorption profile (*λ* = 210 nm) with those of the reference compounds analyzed under the same conditions. For compounds where authentic standards were not available, identification was based on comparison with literature retention data and UV spectral characteristics. The mobile phase was 0.005 N sulfuric acid, flowing at 0.5 mL min^−1^ under an isocratic flow system.^27^

### Sensory analysis

#### Consumer hedonic acceptance tests

The hedonic sensory profiles of *M. sylvestris* L. petal herbal tea infusions were thoroughly assessed using a consumer panel (*n* = 60) following ISO 8586:2023 guidelines.^28^ This panel size and consumer profile are consistent with previous herbal tea research.^11^ This was a hedonic acceptance test (not a paired preference test), as distinct from analytical descriptive analysis. Participants were regular consumers of herbal tea (aged 20–53 years, with equal gender distribution), screened for basic sensory acuity, and naive to the infusion conditions being tested. Prior to evaluation, panelists received a 10 min standardized briefing on the use of the 10 cm unstructured continuous line scale and the definition of each sensory attribute (color, aroma, taste, mouthfeel, aftertaste, overall acceptance), without any reference to the infusion conditions. To minimize bias, non‐visual characteristics were assessed in opaque cups, while color was assessed in transparent cups that replicated normal consumption. All evaluations were conducted in separate sensory booths under controlled conditions to ensure comprehensive and impartial data collection.^29^

### Statistical analysis

All experiments were conducted with at least three independent replicates, and data are presented as mean ± standard deviation (SD). Initial descriptive statistics, including means and SDs, were calculated using Microsoft Excel (Microsoft Corporation, Redmond, WA, USA). For all statistical analyses, the SPSS software package (version 24; IBM Corporation, Armonk, NY, USA), R statistical software (version 3.6.2 (2019‐12‐12) – CRAN), and XLSTAT statistical software (version 2021.2; Addinsoft, New York, NY, USA) were used. For multivariate analyses (principal component analysis (PCA), heatmap, and Spearman correlation), mean values of triplicate measurements represent each infusion condition. This approach reduces the influence of technical variation and focuses on discrimination between the three distinct brewing protocols (4M, 16M, and 24H), consistent with standard practice in multivariate analysis of herbal teas.^6^, ^11^, ^14^

## RESULTS AND DISCUSSION

### Physicochemical properties of mallow (*M. sylvestris* L.) petal herbal teas

The physicochemical properties of *M. sylvestris* petal herbal teas are summarized in Table 1. The dry matter content of the dried petals of *M. sylvestris* L. was 940 g kg^−1^. The pH values of the infusion decreased significantly from 6.23 ± 0.01 (16M) and 6.13 ± 0.06 (4M) to 5.10 ± 0.04 (24H) (*P* < 0.001). Increased release of organic and phenolic acids such as caftaric and chlorogenic acid may cause this acidification in the 24H condition. The relatively lower pH of the 24H condition may contribute to the stabilization of acid‐sensitive antioxidant compounds, as acidic conditions are known to suppress oxidative degradation of phenolics in aqueous systems.^30^, ^31^ Titratable acidity also increased significantly with infusion time (*P* < 0.001), rising from 2.3 ± 0.4 g kg^−1^ (16M) and 3.8 ± 0.4 g kg^−1^ (4M) to 8.5 ± 0.7 g kg^−1^ (24H). This demonstrates that extended cold infusion increases the release of acidic phytochemicals, enhancing their functional properties and storage stability.

**Table 1:** Physicochemical properties of Turkish mallow (*Malva sylvestris* L.) petal herbal teas (mean ± standard deviation)

Parameters	Samples			*P*
	4M	16M	24H	
pH	6.13 ± 0.06^b^	6.23 ± 0.01^c^	5.10 ± 0.04^a^	***
Acidity (g kg^−1^)	3.8 ± 0.4^b^	2.3 ± 0.4^a^	8.5 ± 0.7^c^	***
*Color values*				
L*	79.78 ± 0.01^b^	82.89 ± 0.01^c^	75.94 ± 0.01^a^	***
a*	2.81 ± 0.00^b^	1.44 ± 0.01^a^	8.20 ± 0.01^c^	***
b*	17.62 ± 0.01^a^	18.24 ± 0.00^b^	20.80 ± 0.05^c^	***

*Note:* Mean ± standard deviation: results are the means of three repetitions performed in the laboratory; means in the same row with different superscript letters are significantly different according to Duncan's multiple range test (*P* ≤0.05); *** significant differences at *P* ≤ 0.005. 4M, short hot brewing for 4 min at 98 °C; 16M, extended hot brewing for 16 min at 98 °C; 24H, cold brewing for 24 h at room temperature (25 °C).

Color parameters (L*, a*, b*) also showed significant changes (*P* < 0.001). The L* (lightness) decreased from 82.89 ± 0.01 (16M) and 79.78 ± 0.01 (4M) to 75.94 ± 0.01 (24H), indicating a darker cold‐extracted sample. This is likely because pigmented substances such as flavonoids and anthocyanins are more efficiently extracted and remain stable under cold brewing conditions, as these water‐soluble compounds are known to contribute significantly to the color intensity of herbal infusions and are susceptible to thermal degradation.^32^, ^33^ In contrast to 4M (2.81 ± 0.00) and 16M (1.44 ± 0.01), the a* value (green–red chromaticity) increased significantly in 24H (8.20 ± 0.01), shifting towards red tones, which is consistent with the stability and extractability of anthocyanin derivatives at lower temperatures. The highest b* (blue–yellow axis) value (20.80 ± 0.05) was also observed in 24H.

Overall, these physicochemical changes indicate that the chemical and sensory profile of *M. sylvestris* petal tea is considerably changed by a 24H condition. The darker color, lower pH, and higher acidity for the 24H condition suggest increased extraction of bioactive and pigment compounds, which may enhance the product's functional efficacy and appeal.

### Consumer hedonic perception of mallow (*M. sylvestris* L.) petal herbal teas

A consumer panel (*n* = 60) evaluated the hedonic perception of *M. sylvestris* petal herbal teas in a hedonic acceptance test (not a trained descriptive panel), and the results showed notable variations among different infusion techniques (Table 2 and Fig. 1). Low standard deviations (e.g., 4M color: 8.58 ± 0.51; overall acceptance: 6.86 ± 0.31) and highly significant ANOVA results (*P* < 0.001 for most attributes) confirm the reliability of the consumer panel data. For most sensory attributes, including color (8.58 ± 0.51), taste (6.82 ± 0.56), and overall acceptance (6.86 ± 0.31), the 4M condition consistently received the highest hedonic scores, significantly outperforming other methods (*P* < 0.001). This suggests that compounds contributing to palatability and visual appeal are best extracted by shorter hot brewing times. For aroma perception, the 16M condition scored the lowest (4.16 ± 0.27), while both the 4M (6.54 ± 0.57) and 24H conditions (6.64 ± 0.48) scored significantly higher and did not differ from each other (*P* < 0.001). This indicates that both cold brewing and short hot brewing effectively retain desirable aromatic compounds, whereas prolonged hot brewing degrades them. Additionally, 4M condition was most preferred for taste, followed by 24H, with 16M least preferred, suggesting that extended hot brewing may extract excessive unwanted bitter or astringent compounds. The absence of statistical significance in mouthfeel scores (*P* = 0.094) indicates that infusion method has less impact on this attribute. Aftertaste perception was considerably higher for hot infusions (4M and 16M) than for 24H condition, indicating that hot brewing produces a more tolerable aftertaste.

**Table 2:** Consumer hedonic scores for sensory attributes of Turkish mallow (*Malva sylvestris* L.) petal herbal teas, evaluated by sensory analysis (mean ± standard deviation)

Parameters	Samples			*P*‐Value	ηp^2^
	4M	16M	24H		
Color	8.58 ± 0.51^a^	5.68 ± 0.36^b^	4.39 ± 0.31^c^	**<0.001**	**0.678**
Aroma	6.54 ± 0.57^a^	4.16 ± 0.27^b^	6.64 ± 0.48^a^	**0.001**	**0.409**
Taste	6.82 ± 0.56^a^	3.20 ± 0.18^c^	5.41 ± 0.25^b^	**<0.001**	**0.643**
Mouthfeel	6.46 ± 0.60^a^	5.05 ± 0.32^b^	5.61 ± 0.35^ab^	0.094	0.161
Aftertaste	5.48 ± 0.36^a^	5.22 ± 0.36^a^	3.42 ± 0.35^b^	**0.001**	**0.421**
Overall acceptance	6.86 ± 0.31^a^	4.02 ± 0.20^c^	5.36 ± 0.52^b^	**<0.001**	**0.524**

*Note:* Mean ± standard deviation: values are presented as mean ± standard deviation (*n* = 60 panelists). Means in the same row with different superscript letters are significantly different according to Duncan's multiple range test (*P* ≤ 0.05); ηp^2^, partial eta‐squared effect size. 4M, short hot brewing for 4 min at 98 °C; 16M, extended hot brewing for 16 min at 98 °C; 24H, cold brewing for 24 h at room temperature (25 °C).

**Figure 1:**
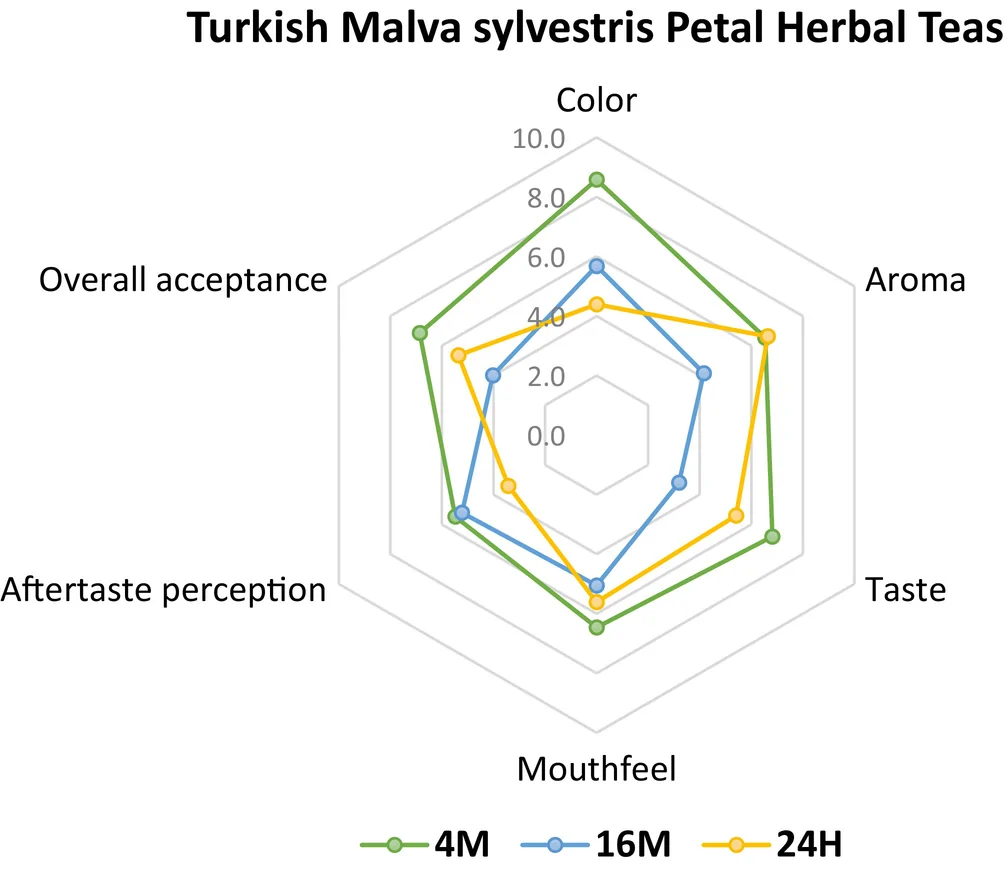
Spider‐web diagram of consumer hedonic scores for sensory attributes of Mallow (*Malva sylvestris* L.) petal herbal teas, evaluated by sensory analysis.

These individual preferences were reflected in the overall acceptance scores, with 4M being the most appealing, followed by 24H, while 16M consistently ranked lowest across most attributes (*P* < 0.001). The importance of infusion parameters in influencing customer satisfaction is highlighted by the significant effect sizes (e.g., ηp^2^ = 0.678 for color, ηp^2^ = 0.643 for taste, and ηp^2^ = 0.524 for overall acceptance). The 4M method produces superior color, aroma, taste, and general acceptability achieving an ideal balance for extracting the desired compounds. In contrast, extended hot infusion (16M) appears detrimental, likely resulting in over‐extraction or degradation. Compared to ideal hot brewing, the 24H condition shows promise in preserving aroma but is less successful in taste and aftertaste. These hedonic findings underscore the importance of optimizing infusion parameters to enhance consumer satisfaction with *M. sylvestris* petal tea and provide essential context for interpreting the detailed physicochemical and phytochemical analyses in the following sections.

### Volatile profile of mallow (*M. sylvestris* L.) petal herbal teas

Herbal tea volatiles arise from various enzymatic processes, such as the lipoxygenase (LOX) pathway for C6 aldehydes and alcohols, the mevalonate (MVA) and methylerythritol phosphate (MEP) pathways for terpenoids, and the shikimate pathway for phenolic volatiles.^34^ As these metabolic pathways are highly sensitive to processing and environmental factors, the volatile composition is significantly influenced by infusion conditions including temperature, duration, and solvent polarity.^6^, ^35^ In this study, the volatile profiles of SAFE volatile extracts from Turkish endemic *M. sylvestris* L. petal herbal teas were analyzed using GC–MS. The extracts were prepared using three different infusion methods: 4M, 16M, and 24H (Table 3).

**Table 3:** Volatile profile of Turkish mallow (*Malva sylvestris* L.) petal herbal teas (mean ± standard deviation)

Number	Volatile compounds (μg L^−1^)	LRI	Samples		
			4 M	16 M	24H
*Acids*					
1	Hexanoic acid	1362	192.9 ± 19.9	n.d.	348.8 ± 31.9
2	Octanoic acid	1570	99.2 ± 11.5	n.d.	213.5 ± 0.0
3	Nonanoic acid	1692	1850.2 ± 183.3	652.3 ± 56.7	2054.5 ± 73.8
4	Decanoic acid	1791	110.6 ± 24.1	n.d.	n.d.
	**Total (4)**		**2252.9**	**652.3**	**2616.8**
*Alcohols*					
5	2‐Methyl‐1‐propanol	931	n.d.	n.d.	60.7 ± 7.0
6	3‐Penten‐2‐ol	1002	35.7 ± 5.2	80.6 ± 51.8	45.8 ± 3.0
7	2,3‐Butanediol	1124	40.0 ± 3.5	n.d.	1131.1 ± 3.0
8	2‐Hexanol	1143	313.8 ± 9.3	398.6 ± 7.3	386.9 ± 4.9
9	Phenethyl alcohol	1507	130.0 ± 0.8	n.d.	276.0 ± 79.0
10	1‐Dodecanol	1588	36.8 ± 0.6	31.6 ± 2.2	48.9 ± 1.1
11	2‐(4‐Methoxyphenyl) ethanol	1809	n.d.	n.d.	193.1 ± 1.1
12	2‐Isopropyl‐5‐methyl‐1‐heptanol	1933	48.7 ± 10.0	35.8 ± 25.3	n.d.
13	1‐Eicosanol	1970	102.7 ± 18.4	44.7 ± 0.0	60.0 ± 0.0
	**Total (9)**		**707.7**	**591.3**	**2202.5**
*Aldehydes*					
14	2‐Methyl pentanal	1071	55.9 ± 3.0	77.6 ± 0.2	73.6 ± 1.4
15	Hexanal	1131	40.3 ± 9.2	64.0 ± 3.5	133.1 ± 0.2
16	2,4‐Dimethyl‐2,4‐heptadienal	1301	n.d.	15.0 ± 1.9	n.d.
17	Nonanal	1489	124.6 ± 39.2	280.2 ± 1.2	275.2 ± 87.6
18	Decanal	1608	45.7 ± 3.6	28.1 ± 0.2	37.3 ± 0.0
19	4‐Propyl‐benzaldehyde	1697	40.0 ± 1.1	n.d.	n.d.
	**Total (6)**		**306.5**	**464.9**	**519.2**
*Esters*					
20	Butyl isobutyrate	1813	145.7 ± 5.9	n.d.	n.d.
21	Diisobutyl phthalate	1869	423.7 ± 24.5	271.3 ± 76.2	274.2 ± 1.5
22	Methyl palmitate	1922	243.5 ± 7.3	156.0 ± 53.2	n.d.
23	Methyl dodecanoate	1974	82.6 ± 20.9	31.4 ± 14.4	n.d.
24	Ethyl palmitate	1987	107.1 ± 6.1	89.3 ± 33.4	n.d.
25	Isopropyl palmitate	2010	53.8 ± 6.7	18.4 ± 6.0	n.d.
	**Total (6)**		**1056.4**	**566.4**	**274.2**
*Ketones*					
26	Acetoin	1027	n.d.	n.d.	1058.2 ± 7.2
27	3‐Hydroxy‐3‐methyl‐2‐butanone	1054	88.3 ± 1.5	111.9 ± 2.9	158.6 ± 2.6
28	4‐Hydroxy‐4‐methyl‐2‐pentanone	1182	41.4 ± 0.6	1012.6 ± 17.8	82.5 ± 0.3
29	Hexahydrofarnesylacetone	1842	n.d.	221.4 ± 30.6	n.d.
	**Total (4)**		**129.7**	**1345.9**	**1299.3**
*Terpenes*					
30	*a*‐Cedrene	1409	31.7 ± 7.7	n.d.	n.d.
31	1,8‐Cineole	1410	13.0 ± 1.1	10.4 ± 0.7	n.d.
32	Carvacrol	1710	1331.4 ± 47.5	532.2 ± 23.1	857.7 ± 27.5
33	Limonene oxide	1794	124.9 ± 9.2	n.d.	n.d.
34	Eugenol	1794	n.d.	n.d.	386.4 ± 7.8
	**Total (5)**		**1501.0**	**542.6**	**1244.1**
*Lactones*					
35	Dihydroactinidiolide	1553	325.9 ± 52.4	226.2 ± 42.5	254.0 ± 27.7
36	𝛾‐Dodecalactone	1800	64.4 ± 0.2	n.d.	n.d.
	**Total (2)**		**390.3**	**226.2**	**254.0**
*Phenols*					
37	4‐Vinyl‐guaiacol	1744	n.d.	n.d.	142.5 ± 0.0
38	2,4‐Di‐*tert*‐butyl phenol	1967	104.0 ± 21.7	67.7 ± 2.0	73.2 ± 19.7
	**Total (2)**		**104.0**	**67.7**	**215.7**
*Benzene*					
39	o‐Xylene	1217	17.5 ± 0.5	41.0 ± 1.4	n.d.
*Furan*					
40	2,3‐Dihydrobenzofuran	1626	95.0 ± 8.3	n.d.	213.6 ± 13.0
	**General total**		**6561.0**	**4498.3**	**8839.4**

*Note:* LRI: linear retention index calculated on DB‐Wax capillary column. Mean ± standard deviation: results are the means of three repetitions performed in the laboratory; n.d., not detected. Totals are shown in bold typeface. 4M, short hot brewing for 4 min at 98 °C; 16M, extended hot brewing for 16 min at 98 °C; 24H, cold brewing for 24 h at room temperature (25 °C).

A GC–MS analysis of the teas identified 40 common volatile compounds (Table 3), categorized as follows: lactones (2), phenols (2), furan (1), alcohols (9), acids (4), aldehydes (6), esters (6), ketones (4), terpenes (5), and phenols (2). There were 33 compounds in 4M, 25 in 16M, and 25 compounds in 24H. Although the overall profiles were similar, infusion parameters (temperature and duration) significantly affected the concentrations of specific compounds and chemical classes. Many identified compounds are also present in *M. sylvestris* essential oil and other herbal teas.^8^, ^36^ The 24H condition had the highest concentration of total volatile (8839.4 μg L^−1^), followed by 4M (6561.0 μg L^−1^) and 16M (4498.3 μg L^−1^), indicating that extended cold infusion improved extraction. In terms of total concentration, the most abundant chemical classes across all infusion conditions were acids (652.3–2616.8 μg L^−1^) and alcohols (591.3–2202.5 μg L^−1^), followed by terpenes (542.6–1501.0 μg L^−1^), ketones (129.7–1345.9 μg L^−1^), esters (274.2–1056.4 μg L^−1^), aldehydes (306.5–519.2 μg L^−1^), lactones (226.2–390.3 μg L^−1^), phenols (67.7–215.7 μg L^−1^), furan (0–213.6 μg L^−1^), and benzene (0–41.0 μg L^−1^) (Table 3). However, the relative abundance of these groups varied among the different brewing methods; for instance, ketones were particularly abundant in the 16M infusion, whereas esters were more prevalent in the 4M sample. Notably, most individual volatile compounds in *M. sylvestris* petal teas were reported for the first time, suggesting that hydrophilic characteristics and aqueous extraction dynamics may have contributed to differences from previous dried petal analyses. Nonanoic acid (652.3–2054.5 μg L^−1^), carvacrol (532.2–1331.4 μg L^−1^), and 2,3‐butanediol (0.0–1131.1 μg L^−1^) were the key compounds with the highest concentrations, although concentrations varied by infusion type. Generally, prolonged hot brewing (16M) increased aldehydes and ketones while decreasing alcohols, acids, esters, terpenes, lactones, and phenols. In contrast, alcohols, acids, aldehydes, ketones, and phenols, increased in 24H, while lactones, terpenes, and esters decreased (Table 3). Similar patterns have been observed in other herbal teas.^6^, ^13^

#### Acids

Depending on chain length and saturation, acids can impart waxy, cheesy, rancid, or sour notes, making them essential contributors to the volatile profile of herbal teas. The conditions of infusion significantly affect their release, which usually results from membrane lipid hydrolysis and oxidation.^6^, ^34^ Four acids, accounting for a substantial proportion of the total volatile compounds (652.3–2616.8 μg L^−1^), were identified in *M. sylvestris* petal herbal teas in this investigation (Table 3). The concentrations of total acids were lowest in 16M (652.3 μg L^−1^), followed by 4M (2252.9 μg L^−1^) and highest in 24H (2616.8 μg L^−1^). This indicates that extended hot infusion (16M) is less effective than prolonged cold infusion, which enhances acid extraction. This is probably because short‐chain acids volatilize or degrade thermally. The most prevalent acid in all samples was nonanoic acid (652.3–2054.5 μg L^−1^), which has been found in other herbal teas and has a fatty, citrusy aroma.^6^ Hexanoic and octanoic acids, which contribute sour and rancid notes, were absent in 16M but present in 4M and 24H, indicating that moderate heat exposure inhibits their stability or release. Only 4M (110.6 μg L^−1^) contained decanoic acid (a waxy, soapy scent), demonstrating the effectiveness of short‐term hot infusion for some thermolabile substances. Overall, the acid profile of *M. sylvestris* petal tea is highly sensitive to infusion parameters; 4M benefits from rapid thermal release, whereas the higher acid content of 24H is associated with passive diffusion and prolonged enzymatic activity. These results are consistent with earlier studies on how temperature and infusion duration affect the composition of fatty acids in herbal teas.^35^ Mallow essential oil has previously been found to contain hexanoic, nonanoic, and decanoic acids;^8^ however, this study is the first to identify octanoic acid.

#### Alcohols

Alcohols, which are often produced by the LOX pathway or enzymatic aldehyde reduction, are common volatile compounds in herbal teas contributing green, floral, fruity, and fermentation notes.^6^, ^34^ At concentrations of 591.3–2202.5 μg L^−1^, they were the second most prevalent volatile group in herbal teas made from *M. sylvestris*. Consistent with earlier studies on oxygenated volatiles, the 24H condition yielded the highest alcohol content (2202.5 μg L^−1^), more than three times that of hot infusions. This suggests that long‐term cold infusion is the most effective method for alcohol extraction.^6^, ^7^ Seven of the nine alcohols identified in *M. sylvestris* petal herbal tea, including 2‐methyl‐1‐propanol, 3‐penten‐2‐ol, and 2,3‐butanediol, are reported here for the first time. 2‐Hexanol, which has a green/herbal scent, was present in large amounts in all types of infusions (313.8–398.6 μg L^−1^). Undetectable in 16M, 2,3‐butanediol increased dramatically to 1131.1 μg L^−1^ in 24H, most likely due to prolonged microbial or enzymatic activity during cold infusion.^37^ The flowery‐tasting phenethyl alcohol also rose markedly in 24H (276.0 μg L^−1^), indicating enhanced release from phenylpropanoid metabolism with extended extraction.^6^ Similarly, 2‐(4‐methoxyphenyl) ethanol (193.1 μg L^−1^) was only present in 24H, indicating that cold infusion is more effective for extracting high molecular weight alcohols.^37^ Although some alcohols have been identified in *M. sylvestris* essential oil^8^ and similar compounds are found in various food items,^38^, ^39^ their profiles in these particular *M. sylvestris* infusions exhibit distinct extraction dynamics.

#### Terpenes

Terpenes are a broad class of volatile compounds that give herbal teas their distinctive woody, minty, citrus, spicy, and floral notes. Produced through the MVA and MEP pathways, they are important contributors to the aroma of *Malva* species^8^, ^40^ and their extraction is extremely sensitive to temperature and time.^34^, ^41^ Total terpene concentrations were lower in 16M (542.6 μg L^−1^), highest in 4M (1501.0 μg L^−1^), and lowest in 24H (1244.1 μg L^−1^). This indicates that terpenes are best extracted by short hot infusion (4M), whereas prolonged heat (16M) results in thermal degradation or volatilization. Similar temperature sensitivity of terpenoids has been observed in *M. silvestris* extracts,^40^ where terpenoid isolation from aqueous extracts was achieved under controlled conditions. Five different terpenes were identified: α‐cedrene, limonene oxide, 1,8‐cineole, carvacrol, and eugenol. Due to its volatility and phenolic nature, carvacrol, which has a spicy/herbal scent,^8^ was the most prevalent (532.2–1331.4 μg L^−1^) and showed higher preservation in short hot (4M) and extended cold (24H) infusions. Low concentrations of 1,8‐cineole (minty notes) were found in hot infusions (10.4–13.0 μg L^−1^), but it was not detectable in 24H, indicating that mild heat exposure during hot brewing may promote its release. The presence of eugenol (a sweet, clove‐like scent) was detected only in 24H (386.4 μg L^−1^), implying that cold infusion improves the extraction of high molecular weight, hydrophobic terpenes.^37^ These results are consistent with studies demonstrating how terpenes in other herbal teas respond to infusion parameters.^6^, ^41^

#### Ketones

Acetoin, 3‐hydroxy‐3‐methyl‐2‐butanone, 4‐hydroxy‐4‐methyl‐2‐pentanone (diacetone alcohol), and hexahydrofarnesylacetone were the four compounds representing the ketone group in the volatile profile of *M. sylvestris* petal tea. Temperature and extraction duration significantly affected ketone release, as shown by the marked variation in their concentrations under different infusion conditions. The 24H condition contained a large amount of acetoin (1058.2 ± 7.2 μg L^−1^), which contributed buttery and creamy notes^12^ and showed enhanced release during prolonged ambient extraction. In contrast, 4‐hydroxy‐4‐methyl‐2‐pentanone peaked in the 16M sample (1012.6 ± 17.8 μg L^−1^), indicating that its release is optimized by intermediate heat exposure. The 4M sample (129.7 μg L^−1^) showed limited extraction efficiency with short infusions, while 16M (1345.9 μg L^−1^) and 24H (1299.3 μg L^−1^) had the highest total ketone content.

#### Esters

Esters are typically formed by esterification reactions between alcohols and fatty acids and are known to contribute fruity, floral, and sweet aroma notes to herbal teas.^12^, ^42^, ^43^ In this study, six esters – butyl isobutyrate, diisobutyl phthalate, methyl hexadecanoate (methyl palmitate), methyl dodecanoate, ethyl hexadecanoate (ethyl palmitate), and isopropyl palmitate were identified in *M. sylvestris* herbal tea infusions (Table 3). Of these, diisobutyl phthalate was the most abundant ester in all infusion types. The 4M sample showed the highest concentration (423.7 ± 24.5 μg L^−1^), followed by 16M (271.3 ± 76.2 μg L^−1^) and 24H (274.2 ± 1.5 μg L^−1^). Although this compound has been associated with packaging‐related contamination in some reports, its consistent presence across all samples may also reflect environmental exposure during processing or storage^12^; however, this interpretation remains tentative and requires further confirmation. The 4M sample had the highest total ester content (1056.4 μg L^−1^), followed by 16M (566.4 μg L^−1^) and 24H (274.2 μg L^−1^). This pattern clearly indicates that lipid‐derived esters are more efficiently extracted by short, high‐temperature infusion, whereas only the more water‐soluble or low molecular weight esters are retained by long ambient infusion.

#### Aldehydes

Aldehydes, commonly produced by the oxidative breakdown of unsaturated fatty acids and the catabolism of amino acids during drying and infusion, are significant contributors to the green, citrus, fatty, and floral aroma notes in herbal teas.^44^, ^45^ In this study, *M. sylvestris* petal tea infusions contained six different aldehydes: 2‐methylpentanal, hexanal, 2,4‐dimethyl‐2,4‐heptadienal, nonanal, decanal, and 4‐propylbenzaldehyde (Table 3). Nonanal was the most prevalent aldehyde across all infusion types, with the highest concentration in the 16M sample (280.2 ± 1.2 μg L^−1^) and similar levels in 24H (275.2 ± 87.6 μg L^−1^) and 4M (124.6 ± 39.2 μg L^−1^). Similarly, nonanal has also been identified as a prominent aldehyde in the petals of *M. sylvestris* L.^8^ and *Malva aegyptiaca* L.^46^ Nonanal is known for its citrus and floral aroma, and it shows an increasing trend with longer infusion times, indicating enhanced release from lipid oxidation products under mild extraction conditions.^6^, ^47^ From 306.5 μg L^−1^ in 4M to 464.9 μg L^−1^ in 16M and 519.2 μg L^−1^ in 24H, the total aldehyde content increased with infusion duration. This trend emphasizes how extended extraction can increase the release of aldehydes, especially those produced by lipid oxidation and amino acid degradation. Nonanal, hexanal, and decanal are present in the 24H and 16M samples, enhancing the olfactory complexity and consumer appeal of tea by contributing to its fresh, citrusy, and slightly fatty aroma. The oxidative stability of the herbal matrix and the impact of infusion parameters on aroma development are also reflected in the aldehyde profile.

#### Lactones

Lactones, which are cyclic esters typically formed from the oxidative breakdown of fatty acids, are well known for imparting fruity, creamy, and woody aroma notes to herbal infusions.^42^, ^43^ Two lactones, dihydroactinidiolide and *γ*‐dodecalactone, were identified in *M. sylvestris* petal herbal tea infusions in the present investigation (Table 3). Of these, dihydroactinidiolide was the predominant lactone in all infusion types; the 4M sample contained the highest concentration (325.9 ± 52.4 μg L^−1^), followed by the 24H sample (254.0 ± 27.7 μg L^−1^) and the 16M sample (226.2 ± 42.5 μg L^−1^). This compound has also been identified in *Camellia sinensis* white tea infusions, where it contributes significantly to the distinctive flavor profile. It is known for its tea‐like, woody, and slightly fruity aroma.^43^ It may be formed during drying and retained during aqueous extraction, given its comparatively high stability across infusion conditions. The 4M sample had the highest total lactone content (390.3 μg L^−1^), followed by the 24H sample (254.0 μg L^−1^) and the 16M sample (226.2 μg L^−1^). This pattern suggests that ambient extraction promotes the retention of more stable lactones, such as dihydroactinidiolide, while short, high‐temperature infusion is more effective at extracting both stable and thermally sensitive lactones.

#### Phenols

The sensory and functional qualities of *M. sylvestris* petal herbal tea are greatly enhanced by phenolic compounds, despite their limited presence in the volatile profile. Two phenolic volatiles were identified in this study: 2,4‐di‐*tert*‐butyl phenol and 4‐vinyl‐guaiacol, which varied significantly depending on the infusion conditions (Table 3). 4‐Vinyl‐guaiacol, a compound with a smoky and clove‐like aroma, was detected only in the 24H sample (142.5 ± 0.0 μg L^−1^), and was absent in the 4M and 16M infusions. Ferulic acid, a hydroxycinnamic acid commonly present in plant cell walls, is typically decarboxylated by heat or microbes to produce this compound.^48^ Its presence in the long‐duration ambient infusion suggests that prolonged contact time may promote mild hydrolytic or enzymatic release from bound precursors, possibly through non‐enzymatic degradation or slow microbial activity. All samples contained 2,4‐di‐*tert*‐butyl phenol, a lipophilic phenolic compound with mild antioxidant properties. The 4M infusion had the highest concentration (104.0 ± 21.7 μg L^−1^), followed by the 24H (73.2 ± 19.7 μg L^−1^) and 16M (67.7 ± 2.0 μg L^−1^) infusions. Rapid solubilization at high temperatures may explain the elevated level in the short hot infusion, whereas oxidative polymerization or thermal degradation may account for the lower values in longer infusions.^49^ The 24H sample had the highest total phenolic volatile content (215.7 μg L^−1^), followed by 4M (104.0 μg L^−1^) and 16M (67.7 μg L^−1^). These findings indicate that a longer infusion time increases the release of phenolic volatiles, which may enhance the tea's functional qualities and sensory complexity. Functionally, phenolic volatiles such as 4‐vinyl‐guaiacol have been associated with antioxidant and antimicrobial properties, which enhance the potential health benefits of herbal teas.^50^

#### Other volatiles

The study found that *o*‐xylene, a benzene derivative with sweet solvent‐like notes, was present in the 4M (17.5 ± 0.5 μg L^−1^) and 16M (41.0 ± 1.4 μg L^−1^) samples but not in the 24H sample. *o*‐Xylene may occur in short‐duration hot infusions due to thermal release from lignin‐derived precursors or slight degradation of aromatic amino acids at high temperatures.^50^ In contrast, the 4M (95.0 ± 8.3 μg L^−1^) and 24H (213.6 ± 13.0 μg L^−1^) samples contained 2,3‐dihydrobenzofuran, a heterocyclic compound with woody and balsamic odor characteristics, while the 16M sample did not contain it. Notably, *o*‐xylene has been previously reported in Tuo tea^51^ and Wuyi rock tea,^52^ while 2,3‐dihydrobenzofuran has been identified in roasted purple corn flour‐based beverages,^53^ supporting the occurrence of both compounds in plant‐based beverage matrices. Both compounds may contribute to the overall depth of aroma and functional character of *M. sylvestris* infusions, despite their low abundance, particularly in formulations aiming for sensory richness and bioactivity.

### Linking aromatic volatile profiles to hedonic sensory perception via PCA, heatmap, and correlation insights

Through an integrated approach combining PCA, heatmap visualization, and correlation analyses with consumer hedonic scores, the complex relationship between brewing conditions and the aromatic volatile profile of *M. sylvestris* petal herbal tea was thoroughly elucidated. PCA was conducted on autoscaled volatile compound concentrations, with the first two principal components accounting for 100% of the total variance (F1: 57.77%, F2: 42.23%; Fig. 2). This analysis revealed clear clustering of volatile compound groups according to infusion techniques. The lactones and esters in Quadrant 1 (Q1) were strongly associated with the 4M infusion. In contrast, the 16M infusion clustered in Quadrant 2 (Q2), which was dominated solely by benzene. Located in Quadrant 3 (Q3), the 24H infusion was characterized by acids, total volatiles, furan, phenols, alcohols, aldehydes, and ketones highlighting the retention of thermolabile, hydrophilic volatiles that produce floral, smoky, and herbal qualities without the use of heat. A heatmap (Fig. 3) provided further information on the varying effects of brewing conditions on volatile compound groups.

**Figure 2:**
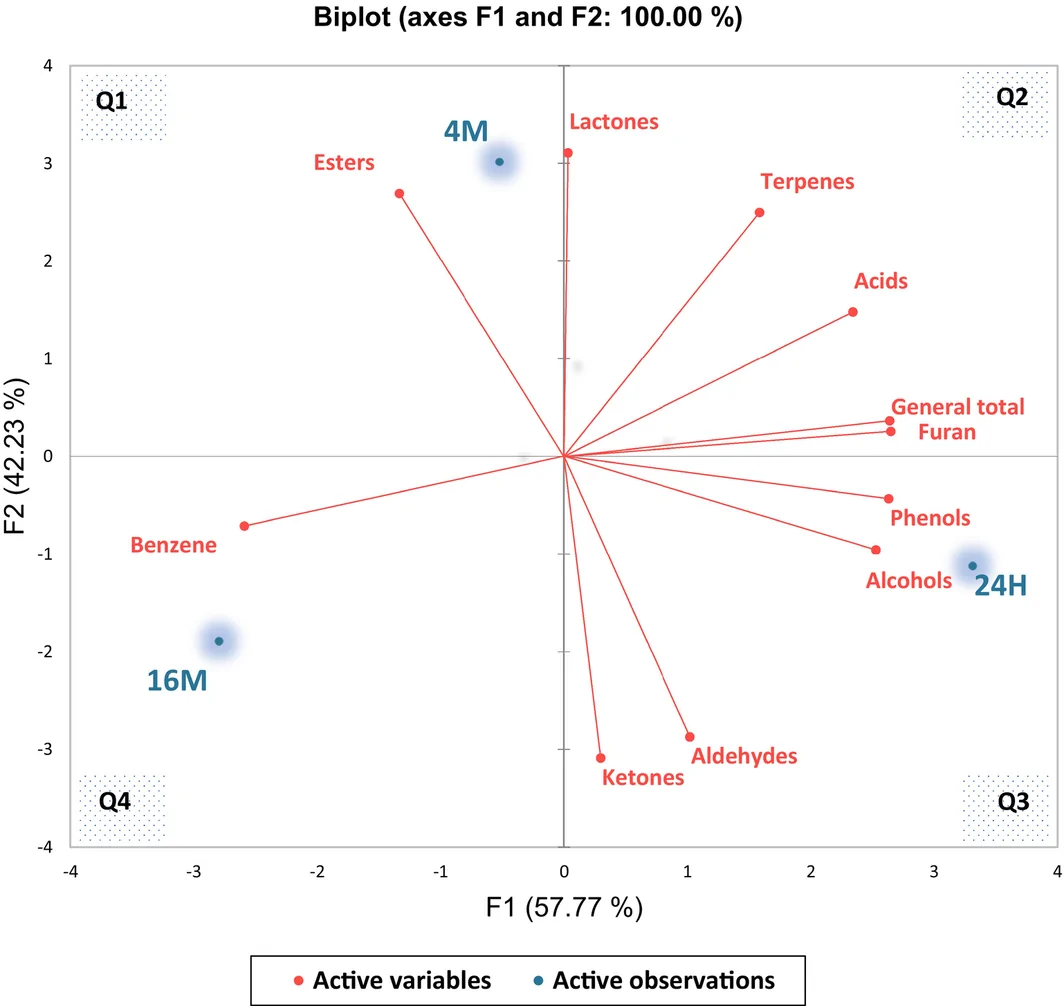
PCA biplot of volatile compounds groups in allow (*Malva sylvestris* L.) petal herbal teas.

**Figure 3:**
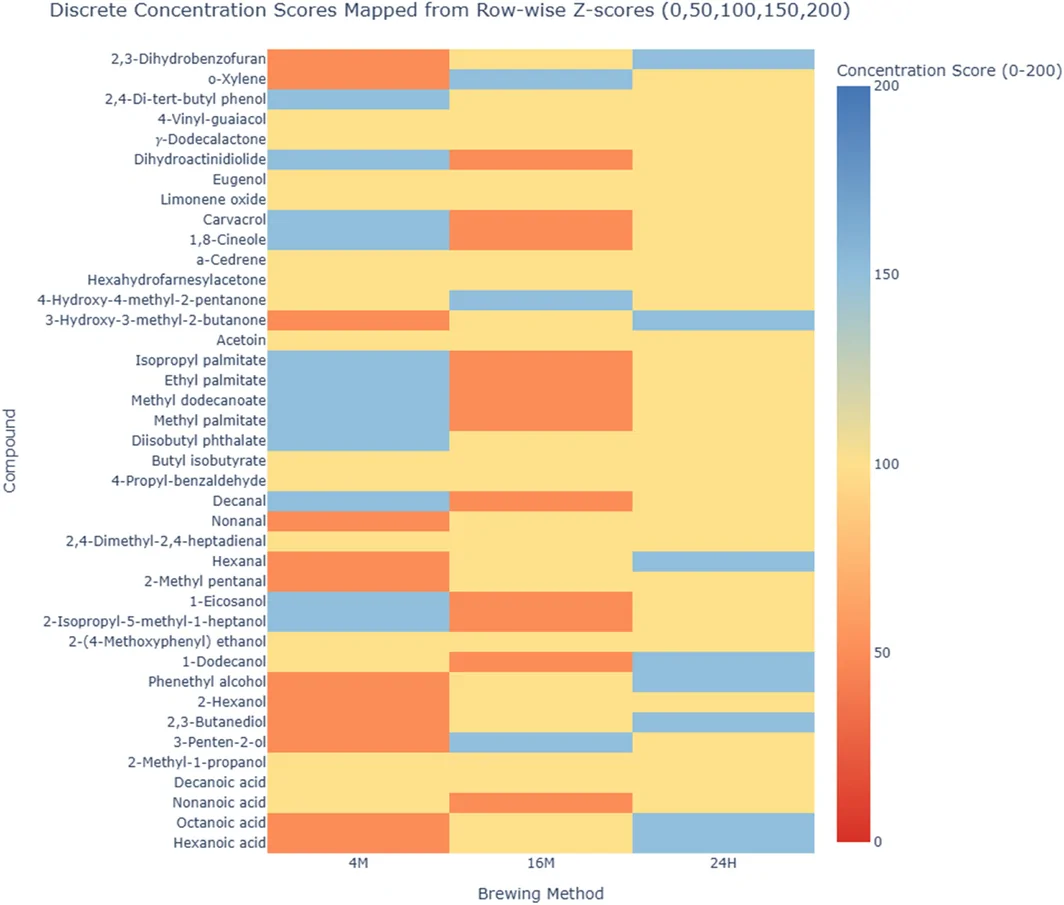
Heatmap of discrete concentration scores for volatile compounds in mallow (*Malva sylvestris* L.) petal herbal tea infusions across different brewing methods.

According to the heatmap visualization of individual compound concentrations, the 4M infusion generally showed higher relative concentrations across a wide range of volatile compounds, especially esters (such as isopropyl palmitate and ethyl palmitate) and long‐chain organic acids (such as decanoic acid), consistent with their rapid extraction during brief hot brewing. Some aldehydes and ketones remained at moderate levels, which is consistent with the PCA results, although the 16M infusion often showed intermediate to lower concentrations for many of these compounds, indicating thermal degradation or volatilization. However, as PCA revealed, the 24H condition maintained a distinct profile of acids, total volatiles, furans, phenols, alcohols, aldehydes, and ketones, indicating different extraction efficiencies or selective retention under cold conditions. In contrast, the infusion usually showed lower relative concentrations for many volatiles.

Given the limited number of infusion conditions (*n* = 3), correlation analyses between volatile compounds and hedonic sensory attributes were performed solely for exploratory purposes and should not be interpreted as statistically robust relationships. For this analysis, the mean concentration of each volatile compound was correlated with the mean sensory scores for each attribute (color, aroma, taste, mouthfeel, aftertaste, and overall acceptance) obtained from the consumer panel (*n* = 60). Data distribution was assessed using the Shapiro–Wilk test, which indicated non‐normality for most variables; therefore, Spearman's rank correlation coefficient (rho) was used as the appropriate non‐parametric measure. A positive rho value indicates that higher concentrations of a volatile compound are associated with higher sensory scores, while a negative rho suggests the opposite.

Spearman correlation analyses revealed consistent directional trends between specific volatiles and sensory attributes (Fig. 4). For illustrative purposes, compounds with the strongest correlation coefficients (rho ≈ 1.0) and consistent directional trends across the three key hedonic attributes (‘Aroma,’ ‘Taste,’ and ‘Overall Acceptance’) were highlighted. For example, nonanoic acid, ethyl palmitate, isopropyl palmitate, and methyl palmitate showed strong positive associations (rho ≈ 1.0) with ‘Aroma,’ ‘Taste,’ and ‘Overall Acceptance.’ This is consistent with the higher abundance of these compounds in the 4M infusion, which also received the highest hedonic scores for these attributes. Conversely, 3‐penten‐2‐ol, 2‐methyl‐1‐propanol, and phenethyl alcohol displayed strong negative associations (rho ≈ −1.0) with favorable sensory scores, suggesting possible contributions to off‐notes during prolonged extraction. Although these patterns provide heuristic insights into aroma‐perception relationships, they require validation in future studies with larger experimental designs.

**Figure 4:**
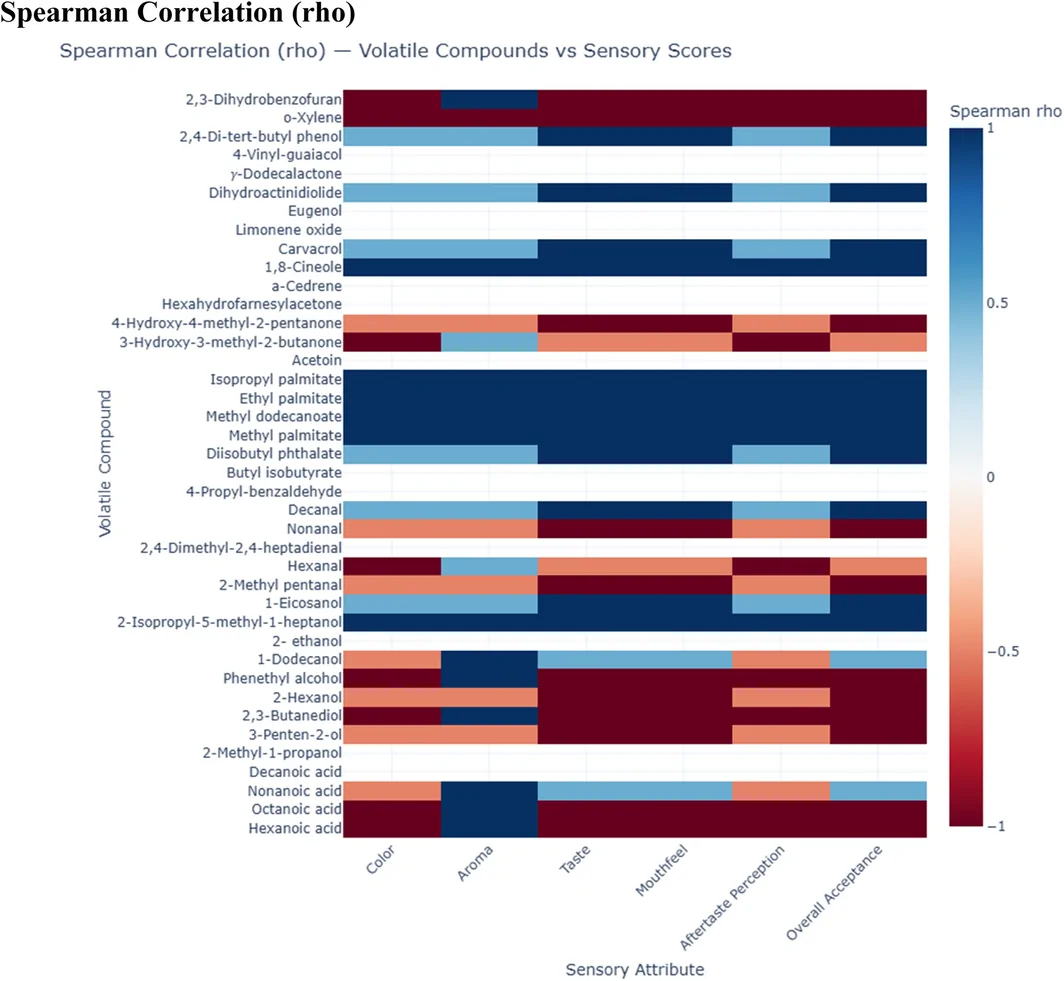
Spearman correlation (rho) between volatile compounds and hedonic sensory attributes of mallow (*Malva sylvestris* L.) petal herbal tea infusions.

### Phenolic profile of mallow (*M. sylvestris* L.) petal herbal teas

One of the most physiologically active phytochemicals, phenolic compounds are well known for their antibacterial, anti‐inflammatory, and antioxidant properties in both edible and medicinal plants.^3^, ^54^ When taken as a herbal tea or in functional foods, *M. sylvestris* is especially high in these compounds.^4^ The phenolic compounds were classified into four main categories: phenolic acids, flavonoids, stilbenes, and tannins, to provide a comprehensive understanding of the phytochemical dynamics (Table 4). Each group was evaluated separately, with a focus on statistically significant variations among infusion conditions and comparison with previously published studies. A comprehensive quantitative analysis (Table 4) showed that the 24H condition significantly enhanced phenolic extraction compared to both hot brewing methods (4M and 16M). Total phenolic content increased from 45.92 mg L^−1^ (4M) and 38.15 mg L^−1^ (16M) to 79.39 mg L^−1^ (24H), while total flavonoids increased from 29.17 mg L^−1^ (4M) and 23.36 mg L^−1^ (16M) to 57.82 mg L^−1^ (24H). Notably, the 16M condition yielded the lowest total phenolic and flavonoid contents among all samples. This observation can be attributed to two main factors: extraction time and temperature. First, the short infusion times (4 and 16 min) limited the release of phenolic compounds compared to the 24‐h cold infusion, which allowed prolonged contact between the plant matrix and the aqueous medium, facilitating passive diffusion and enhanced solubilization of water‐soluble phenolics. Second, the high temperature (98 °C) applied during hot brewing may have promoted thermal degradation or oxidative polymerization of thermolabile phenolic compounds, particularly in the 16M sample where prolonged heat exposure exacerbated these effects. In contrast, the 4M condition provided a moderate phenolic yield while better preserving heat‐sensitive compounds due to the shorter heating period, whereas the 24H condition, performed at room temperature (25 °C) without thermal stress, was the most effective for extracting total phenolic and total flavonoid contents. To assess the functional implications, this section also briefly correlates phenolic content with antioxidant activity (DPPH and ABTS).

**Table 4:** Phenolic profile and antioxidant activities of Turkish mallow (*Malva sylvestris* L.) petal herbal teas (mean ± standard deviation)

Number	Phenolic compounds (mg L^−1^)	Samples			*P*
		4M	16M	24H	
*Phenolic acids*					
1	Gallic acid	0.04 ± 0.00	0.07 ± 0.04	0.07 ± 0.00	NS
2	Protocatechuic acid	0.02 ± 0.00^a^	0.01 ± 0.00^a^	0.12 ± 0.01^b^	***
3	2,4‐Dihydroxy benzoic acid	0.16 ± 0.03^a^	0.11 ± 0.04^a^	0.27 ± 0.01^b^	*
4	Syringic acid	0.23 ± 0.06^a^	0.13 ± 0.03^a^	0.60 ± 0.01^b^	**
5	Caftaric acid	2.79 ± 0.17^b^	1.99 ± 0.34^a^	3.86 ± 0.05^c^	**
6	Chlorogenic acid	2.48 ± 0.12^a^	2.21 ± 0.11^a^	3.20 ± 0.00^b^	**
7	2,5‐Dihydroxy benzoic acid	3.78 ± 0.34^a^	3.11 ± 0.21^a^	5.58 ± 0.01^b^	**
8	*t*‐Caffeic acid	0.62 ± 0.12^a^	0.45 ± 0.20^a^	1.69 ± 0.36^b^	*
9	*p*‐Coumaric acid	1.91 ± 0.01^a^	1.89 ± 0.01^a^	1.99 ± 0.00^b^	**
10	Sinapic acid	1.45 ± 0.00^b^	1.44 ± 0.00^a^	1.47 ± 0.00^c^	**
11	Ferulic acid	1.67 ± 0.01^b^	1.63 ± 0.01^a^	1.68 ± 0.00^b^	NS
	**Total phenolic acids**	**15.15**	**13.04**	**20.53**	
*Flavonoids*					
12	Catechin	7.35 ± 1.20^a^	5.07 ± 0.84^a^	14.16 ± 0.02^b^	**
13	Epicatechin	4.08 ± 0.65^a^	2.81 ± 0.40^a^	7.58 ± 0.05^b^	**
14	Rutin	9.22 ± 2.72^a^	7.19 ± 0.98^a^	20.12 ± 0.24^b^	**
15	Quercetin‐3‐glucoside	2.03 ± 0.18	1.83 ± 0.13	2.03 ± 0.08	NS
16	Kaempferol‐3‐glucoside	1.00 ± 0.03^b^	0.94 ± 0.01^a^	1.20 ± 0.00^c^	***
17	Myricetin	1.46 ± 0.03^ab^	1.41 ± 0.02^a^	1.48 ± 0.00^b^	NS
18	Quercetin	0.95 ± 0.00^a^	0.95 ± 0.00^a^	1.11 ± 0.00^b^	***
19	Luteolin	2.07 ± 0.17^a^	2.14 ± 0.14^a^	8.51 ± 0.11^b^	***
20	Kaempferol	1.01 ± 0.02^a^	1.02 ± 0.01^a^	1.63 ± 0.01^b^	***
	**Total flavonoids**	**29.17**	**23.36**	**57.82**	
*Stilbene*					
21	Resveratrol	0.20 ± 0.00	0.20 ± 0.00	0.21 ± 0.00	NS
*Tanin*					
22	Procyanidin B2	1.40 ± 0.15^b^	1.55 ± 0.16^b^	0.83 ± 0.03^a^	*
	**Total phenol**	**45.92**	**38.15**	**79.39**	
*Antioxidant activity (mg Trolox equivalent kg* ^*−1*^)					
	DPPH	352.62 ± 4.55	348.69 ± 1.90	342.22 ± 22.51	NS
	ABTS	75.38 ± 0.33^a^	77.52 ± 0.22^b^	77.71 ± 0.18^b^	**

*Note:* Mean ± standard deviation: results are the means of three repetitions performed in the laboratory; means in the same row with different superscript letters are significantly different according to Duncan's multiple range test (*P* ≤0.05); *, ** and *** significant differences at *P* ≤ 0.05. *P* ≤ 0.01 and *P* ≤ 0.005, respectively; NS, not significant. Totals are shown in bold typeface. 4M, short hot brewing for 4 min at 98 °C; 16M, extended hot brewing for 16 min at 98 °C; 24H, cold brewing for 24 h at room temperature (25 °C).

Among the individual phenolic acids, the concentration of 2,5‐dihydroxybenzoic acid was consistently the highest across all infusion types, with statistically significant differences between groups (*P* < 0.01). During the 24H condition, it peaked at 5.58 ± 0.01 mg L^−1^, demonstrating its significant contribution to the phenolic profile. It is well known that this compound enhances the overall antioxidant capacity of herbal infusions.^54^ Caftaric acid, another major hydroxycinnamic acid in *M. sylvestris* petal tea infusions, also showed high concentrations, reaching 3.86 ± 0.05 mg L^−1^ in the 24H infusion. This value confirms caftaric acid as a major hydroxycinnamic acid in *M. sylvestris* infusions and represents a 38% increase over the 4M infusion. According to earlier research, caftaric acid is one of the main phenolic acids found in *M. sylvestris* leaves and flowers, and it also significantly increases the antioxidant capacity of plant.^3^, ^4^, ^54^ Another well‐known hydroxycinnamic acid, chlorogenic acid, significantly increased (*P* < 0.01) from 2.48 ± 0.12 mg L^−1^ (4M) to 3.20 ± 0.00 mg L^−1^ (24H). Chlorogenic acid was initially identified in *Malva* extracts and has been linked to anti‐inflammatory and free radical scavenging properties.^4^ The increase from 15.15 mg L^−1^ (4M) to 20.53 mg L^−1^ (24H) in total phenolic acids demonstrates the effectiveness of prolonged aqueous extraction in releasing bioactive compounds from the floral matrix. While previous research^3^, ^4^ has verified the presence of phenolic acids in *M. sylvestris* flowers and leaves, the specific impact of infusion duration on their extraction has not been extensively studied. The current findings provide new insight into time‐dependent extraction dynamics, suggesting that prolonged cold infusion may be an optimized technique for enhancing the recovery of antioxidant‐active phenolics from *M. sylvestris* floral tissues.

Flavonoids are well known for their potent antioxidant, cytoprotective, and anti‐inflammatory properties.^3^, ^54^, ^55^ The effectiveness of long‐term cold infusion was confirmed by the nearly two‐fold increase in total flavonoid content in 24H (57.82 mg L^−1^) compared to 4M (29.17 mg L^−1^) and 16M (23.36 mg L^−1^), with significant differences across all groups (*P* < 0.01). The predominant individual flavonoid in 24H was rutin (20.12 ± 0.24 mg L^−1^, *P* < 0.01), which possess antioxidant and vascular‐protective qualities.^3^ The sensitivity of catechin and epicatechin to infusion duration and their role in radical scavenging activity^4^ was confirmed by their notable increases in the 24H infusion, reaching 14.16 ± 0.02 mg L^−1^ and 7.58 ± 0.05 mg L^−1^, respectively (*P* < 0.01). Luteolin, which has strong anti‐inflammatory properties,^3^ increased significantly from 2.07 ± 0.17 mg L^−1^ (4M) to 8.51 ± 0.11 mg L^−1^ (24H, *P* < 0.001). Significant increases were also observed in quercetin, kaempferol, and their glycosides during the 24H infusion. For example, quercetin increased from 0.95 ± 0.00 mg L^−1^ (4M and 16M) to 1.11 ± 0.00 mg L^−1^ (24H) (*P* < 0.001), and kaempferol from 1.01 ± 0.02 mg L^−1^ (4M) and 1.02 ± 0.01 mg L − 1 (16M) to 1.63 ± 0.01 (24H) (*P* < 0.001). There were no significant differences between myricetin and quercetin‐3‐glucoside (*P* > 0.05). Overall, the extraction of flavonoids, particularly flavonol glycosides and flavones, which are essential for the antioxidant and therapeutic potential of *M. sylvestris* petal infusions, was significantly enhanced by the 24H condition, providing new insight into the dynamics of time‐dependent flavonoid extraction.

Resveratrol, a stilbene, was detected in all *M. sylvestris* petal infusions at comparable levels (0.20–0.21 mg L^−1^), with no statistically significant differences (*P* > 0.05). This stability is likely due to its limited solubility and structural resilience, which suggest insensitivity to aqueous extraction time. Although present at low concentrations, resveratrol is well‐known for its chemopreventive, cardioprotective, and anti‐inflammatory properties. Its presence contributes to the broader therapeutic profile of *M. sylvestris* infusions.^3^

Procyanidin B2, a member of the tannin group, showed a significant decrease in the 24H sample (0.83 ± 0.03 mg L^−1^) compared to shorter infusions (1.40 ± 0.15 mg L^−1^ in 4M, 1.55 ± 0.16 mg L^−1^ in 16M) (*P* < 0.05). Polymerization reactions or hydrolytic degradation due to prolonged aqueous exposure may account for this reduction. However, procyanidin B2 continues to play a vital role in the antioxidant capacity of *M. sylvestris*, particularly in short‐duration infusions where its stability is better preserved.

### Antioxidant activities of mallow (*M. sylvestris* L.) petal herbal teas

Antioxidant activity, a key functional characteristic of herbal infusions, reflects their capacity to combat free radicals and reduce oxidative stress, and was assessed in *M. sylvestris* petal infusions using both ABTS and DPPH assays (Table 4). Antioxidant activity increased significantly from 75.38 ± 0.33 mg Trolox equivalent kg^−1^ (4M) to 77.71 ± 0.18 mg Trolox equivalent kg^−1^ (24H) (*P* < 0.01) in the ABTS assay, while no statistically significant differences were observed between infusion conditions in the DPPH assay. This apparent discrepancy can be explained by several interrelated factors. Although both assays are generally classified as electron transfer (ET)‐based methods, they differ in their dominant reaction pathways and reactivity toward phenolic structures.^56^, ^57^ Moreover, the 24H condition was characterized by a significant increase in specific phenolic acids and flavonoids, as determined by HPLC (Table 4). The structural features of these compounds, such as hydroxylation pattern and conjugation, are known to influence their radical scavenging activity.^58^ Furthermore, prolonged cold extraction may facilitate the solubilization of polar compounds that are more reactive to ABTS, while short hot infusions may generate Maillard reaction products that contribute to DPPH scavenging independently of phenolic content.^59^ Notably, the 16M infusion, despite having the lowest total phenolic content, exhibited DPPH activity comparable to the 24H infusion, further supporting the critical role of phenolic composition in assay‐specific antioxidant activity.^58^ Overall, both the quantity and the chemical nature of phenolic compounds determine the antioxidant capacity, and assay selection is crucial for accurately evaluating the functional potential of herbal infusions.

### Phenolic compounds and antioxidant activity: integrated PCA and correlation insights

The phenolic profile and antioxidant capacity of *M. sylvestris* petal herbal tea were significantly influenced by the different brewing techniques; this was thoroughly investigated using PCA (Fig. 5) and correlation analyses (Fig. 6). PCA was applied to autoscaled phenolic and antioxidant activity data, with the first two components explaining 100% of the cumulative variance (F1: 85.73%, F2: 14.27%; Fig. 5). This analysis clearly separated phenolic compounds and antioxidant activities into four quadrants. The 24H condition was strongly associated with Q1, characterized by high concentrations of numerous phenolic acids (e.g., kaempferol, caftaric, chlorogenic, 2,5‐dihydroxybenzoic, syringic, sinapic acids, and others), total phenolic content, and ABTS antioxidant activity. This indicates that prolonged cold infusion effectively extracts a broad spectrum of hydrophilic phenolics. The 16M condition was linked to Q2, showing a strong correlation with procyanidin B2. The 4M condition was positioned in Q3, showing a clear association primarily with DPPH antioxidant activity. Quadrant 4 (Q4) showed no direct association with any specific infusion condition or primary active variable in this biplot. These PCA results clearly demonstrate that both temperature and infusion duration are critical determinants of the phenolic composition and antioxidant potential of the studied teas, with 24H condition optimizing the recovery of a wide range of bioactive phenolics and overall antioxidant capacity.

**Figure 5:**
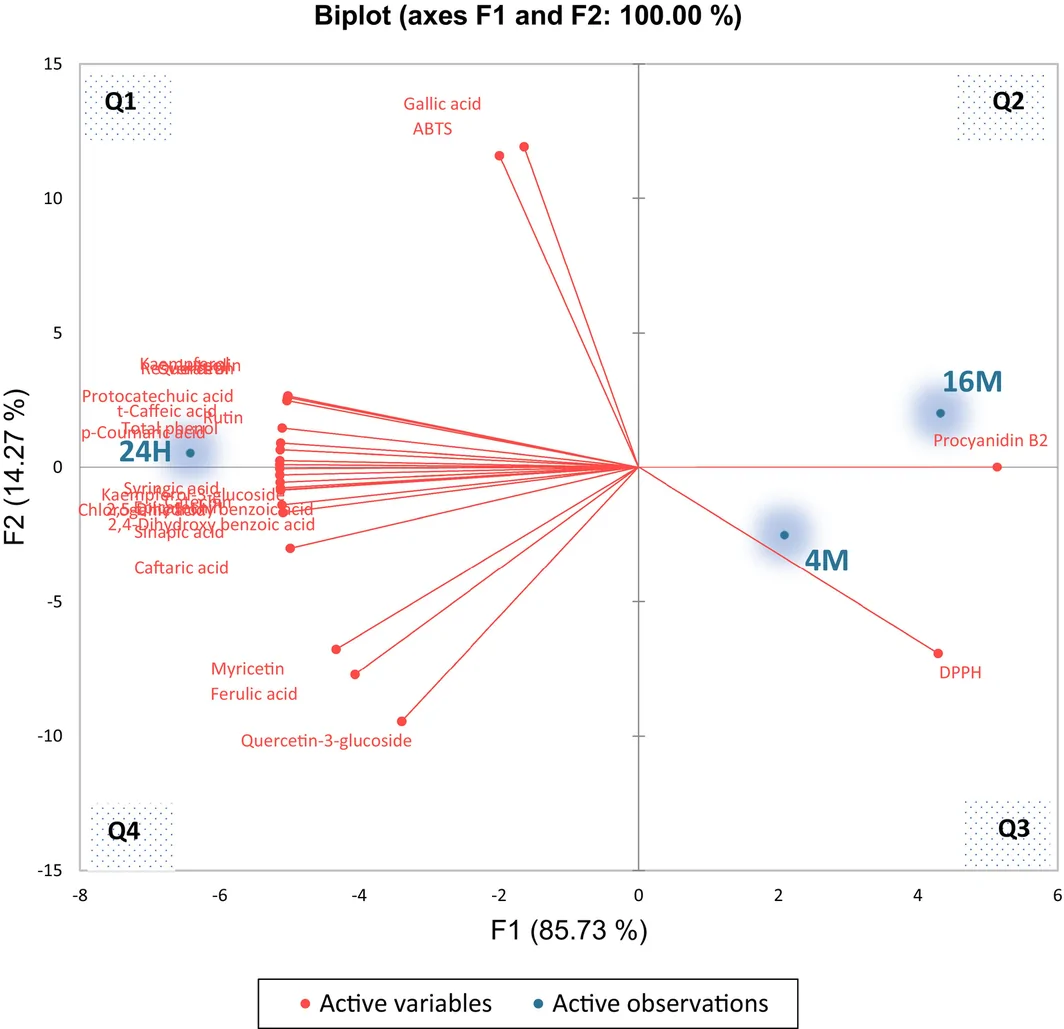
PCA biplot of phenolic compounds in mallow (*Malva sylvestris* L.) petal herbal teas.

**Figure 6:**
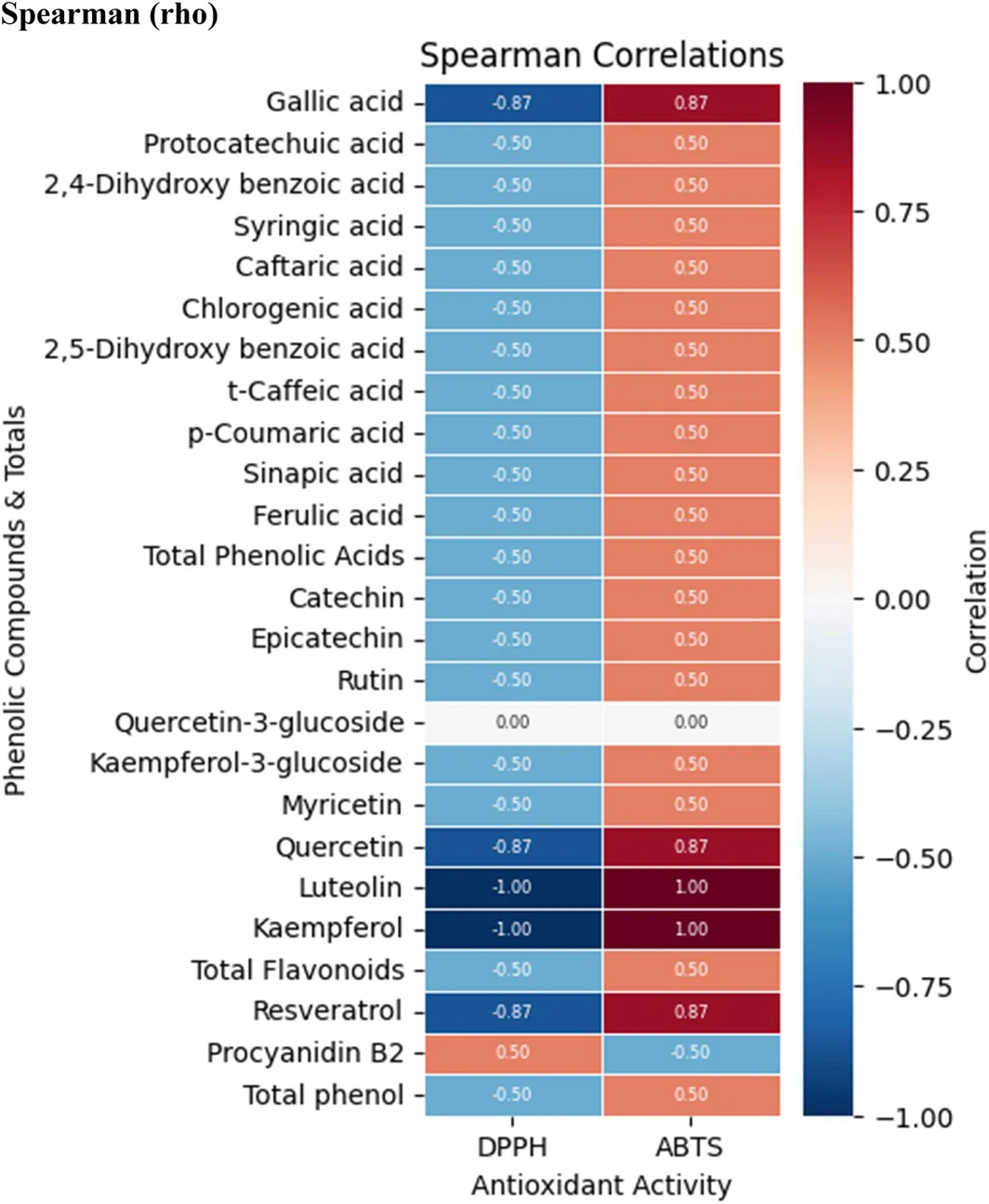
Spearman correlation (rho) between phenolic compounds and antioxidant activities of mallow (*Malva sylvestris* L.) petal herbal tea infusions.

Correlation analyses between phenolic compounds and antioxidant activities were conducted with caution due to the limited number of infusion conditions (*n* = 3), which restricts statistical power. The Shapiro–Wilk test confirmed a non‐normal distribution for most phenolic and antioxidant response variables; consequently, Spearman's rank correlation coefficient (rho) was used to evaluate monotonic relationships.

Spearman correlation analyses (Fig. 6) showed that gallic acid, protocatechuic acid, total phenolic acids, and total phenol had strong positive associations (rho ≈ 0.87–1.00) with ABTS radical scavenging activity. In contrast, luteolin, kaempferol, and rutin had strong negative associations (rho ≈ −1.00) with DPPH response, reflecting assay‐specific reactivity differences among flavonoid subclasses. Procyanidin B2 showed a moderate positive association with DPPH (rho = 0.50), consistent with its known hydrogen‐donating capacity. These trends support the PCA‐based clustering and highlight the differential contributions of phenolic subclasses to antioxidant mechanisms, though confirmation in expanded experimental frameworks is still necessary.

### Organic acids of mallow (*M. sylvestris* L.) petal herbal teas

Eight organic acids, which largely influence the acidity, taste, and metabolic importance of herbal teas obtained from the petals of *M. sylvestris*, were determined by HPLC, revealing statistically significant differences among the infusion conditions (Table 5). In particular, the 24H condition significantly increased the concentration of most organic acids compared to the 4M and 16M conditions (*P* < 0.001 or *P* < 0.01). For example, citric acid (430.75 ± 2.69 mg L^−1^ in 24H), resulting from the enhanced solubility of cell wall and vacuole‐associated citrates^60^; tartaric acid (47.81 ± 11.06 mg L^−1^ in 24H), reflecting time‐dependent release processes associated with intracellular diffusion; malic acid (151.12 ± 0.66 mg L^−1^ in 24H), which accumulated due to the hydrolysis of esterified forms or increased permeability of parenchymal tissues^61^; succinic acid (214.86 ± 46.52 mg L^−1^ in 24H), which may arise from secondary metabolic activity or non‐enzymatic decarboxylation^60^; and lactic acid (438.59 ± 48.71 mg L^−1^ in 24H), an indicator of potential anaerobic metabolic activity or mild spontaneous fermentation,^61^ were found to be significantly higher in the 24H infusion. Similarly, the most common short‐chain fatty acid, acetic acid (1136.13 ± 80.30 mg L^−1^ in 24H), as well as propionic acid (1409.41 ± 15.25 mg L^−1^ in 24H) and butanoic acid (585.65 ± 27.18 mg L^−1^ in 24H) also increased in the 24H condition; these results may indicate microbial acetogenesis, the hydrolysis of acetylated compounds, or mild fermentation. Overall, the 24H condition significantly enhanced the extraction of all organic acids by increasing their release through hydrolysis, microbial transformation, or passive diffusion; these findings are consistent with previous studies on *M. sylvestris*.^9^, ^60^, ^61^

**Table 5:** Organic acids profile of Turkish mallow (*Malva sylvestris* L.) petal herbal teas (mean ± standard deviation)

Number	Organic acids (mg L^−1^)	Samples			*P*
		4M	16M	24H	
1	Citric acid	141.79 ± 12.45^b^	86.33 ± 15.39^a^	430.75 ± 2.69^c^	***
2	Tartaric acid	10.67 ± 3.45^a^	7.72 ± 0.40^a^	47.81 ± 11.06^b^	*
3	Malic acid	51.27 ± 4.55^b^	18.50 ± 2.31^a^	151.12 ± 0.66^c^	***
4	Succinic acid	38.38 ± 1.62^a^	27.89 ± 0.98^a^	214.86 ± 46.52^b^	**
5	Lactic acid	65.55 ± 3.03^a^	35.92 ± 4.41^a^	438.59 ± 48.71^b^	***
6	Acetic acid	444.31 ± 20.45^b^	186.18 ± 6.32^a^	1136.13 ± 80.30^c^	***
7	Propionic acid	144.23 ± 25.25^b^	43.12 ± 10.25^a^	1409.41 ± 15.25^c^	***
8	Butanoic acid	179.41 ± 32.65^b^	94.30 ± 2.63^a^	585.65 ± 27.18^c^	***

*Note:* Mean ± standard deviation: results are the means of three repetitions performed in the laboratory; means in the same row with different superscript letters are significantly different according to Duncan's multiple range test (*P* ≤0.05); *. ** and *** significant differences at *P* ≤ 0.05. *P* ≤ 0.01 and *P* ≤ 0.005, respectively. 4M, short hot brewing for 4 min at 98 °C; 16M, extended hot brewing for 16 min at 98 °C; 24H, cold brewing for 24 h at room temperature (25 °C).

For the PCA results of the organic acids, based on their organic acid profiles, they were distributed across the four quadrants (Q1–Q4), showing that *M. sylvestris* herbal tea infusions were clearly separated by PCA (Fig. 7). During the short hot infusion, Q1 (4M) was associated with lower concentrations of succinic and citric acids, indicating limited thermal extraction. The higher levels of fumaric and malic acids in Q2 (16M) suggest that prolonged heating promotes the release of specific acids by breaking down the matrix more thoroughly. As tartaric and oxalic acids are better preserved under cold infusion conditions, Q3 (24H) exhibited the highest total organic acid content. These compounds contribute to the tea's fresh, sour, and mildly astringent flavor. The intermediate extraction behavior was reflected in Q4, which showed transitional profiles with overlapping acid markers. The organic acid composition of *M. sylvestris* tea is greatly influenced by infusion time and temperature, according to the overall PCA results. Cold infusion allows better retention of sensitive and hydrophilic acids.

**Figure 7:**
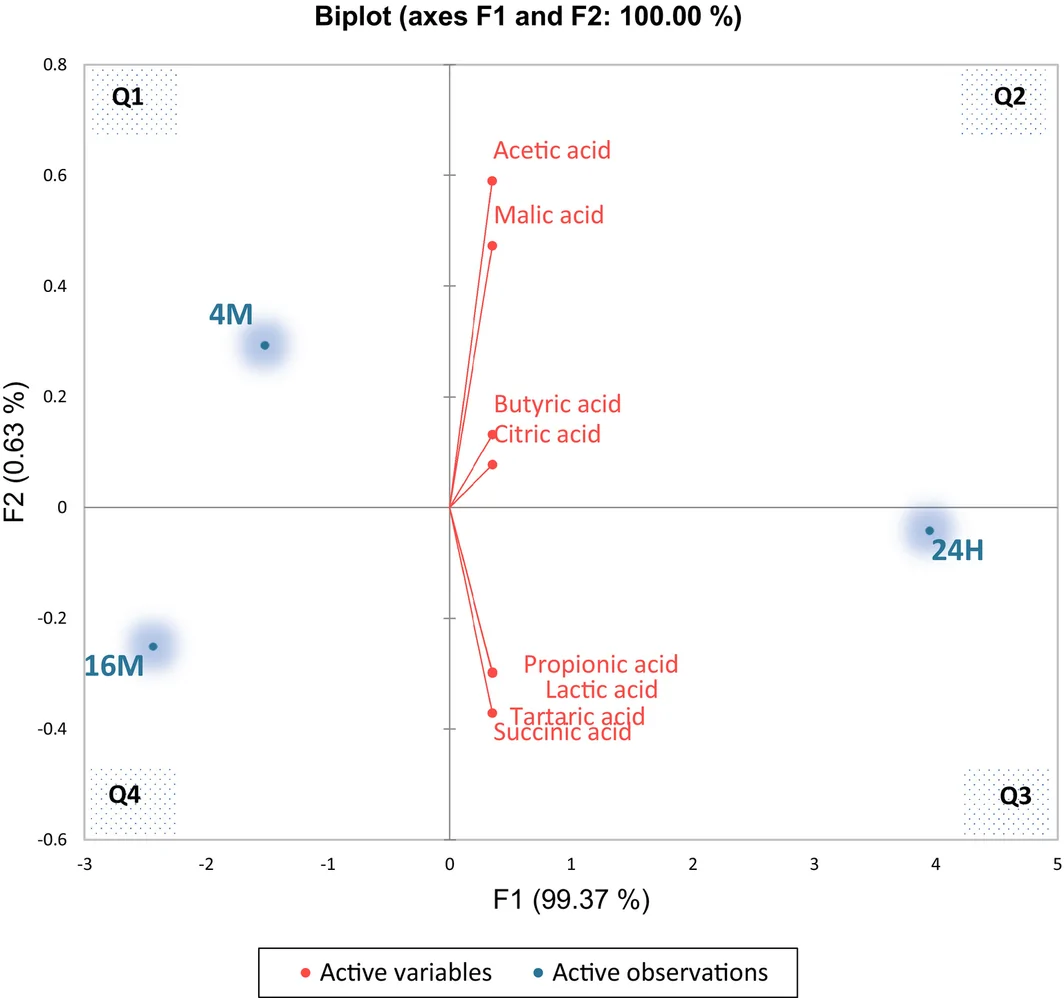
PCA biplot of organic acids in mallow (*Malva sylvestris* L.) petal herbal teas.

## CONCLUSION

This study provides the first integrated characterization of *M. sylvestris* L. petal tea under three consumption‐relevant brewing protocols, 4M, 16M, and 24H, linking extraction dynamics to phytochemical profiles, antioxidant functionality, and sensory perception. Although the 24H method is consumed in some traditional context, its lower hedonic scores (Table 2) indicate that it is not sensorially optimal for daily consumption. However, despite its poorer sensory performance, this method may still be preferable from a health perspective, as it yielded higher levels of phenolic compounds, flavonoids, and tannins, along with relevant antioxidant activity suggesting potential functional benefits. The 16M method, in contrast, yielded the lowest total phenolic and flavonoid contents among all samples, which can be attributed to prolonged heat exposure promoting thermal degradation or oxidative polymerization of thermolabile phenolic compounds. Moreover, the 16M infusion exhibited DPPH activity comparable to the 24H infusion despite its lower phenolic content, suggesting that the qualitative phenolic composition, rather than total quantity, plays a critical role in determining assay‐specific antioxidant activity. The results show that infusion conditions act as a critical modulator of tea quality: short hot infusion best preserves volatile esters and terpenes responsible for sweet, floral, and woody notes, yielding the highest consumer acceptance; cold brewing maximizes the extraction of water‐soluble phenolics (e.g., gallic acid, sinapic acid) and organic acids, yielding the highest total phenolic content and ABTS radical scavenging activity; while extended hot infusion promotes lipid oxidation products (e.g., nonanal) and partial degradation of thermolabile antioxidants, leading to compromised sensory and functional attributes.

This investigation focused on three representative brewing scenarios commonly encountered in domestic and artisanal practice, capturing key extremes of time–temperature regimes relevant to real‐world consumption. While this approach enables direct applicability to traditional use and product development, future studies using continuous kinetic profiling or multi‐regional plant material could further refine the understanding of compound release dynamics and chemotypic variability. Similarly, although chemical antioxidant assays provide valuable initial insights, complementary *in vitro* digestion models would enhance the physiological relevance of the observed bioactivities. The mechanistic explanation for the ABTS *versus* DPPH discrepancy, including the role of phenolic structural features, Maillard reaction products, and assay‐specific reactivity, should be further validated in future research using time‐resolved kinetic profiling and targeted compound analysis. Sensory evaluation was based on hedonic consumer scoring; integrating descriptive analysis or aroma‐active compound identification (e.g., via gas chromatography–olfactometry) in future work would strengthen the link between molecular composition and perceptual experience.

From an applied perspective, these findings support the strategic selection of infusion protocols based on desired outcomes: short hot infusion for sensory‐driven applications, and cold brewing for functional beverage development targeting antioxidant enrichment. The 16M condition is not recommended for either sensory or functional purposes. This work establishes a methodological foundation for the evidence‐based valorization of underutilized floral resources in natural product innovation, aligning ethnobotanical tradition with contemporary demands for sustainable, health‐promoting foods and beverages. This study provides fundamental information for maximizing the health‐promoting qualities and sensory appeal of tea prepared from *M. sylvestris* L. A key strength of this work is the analysis of samples prepared in consumption‐relevant forms, ensuring the direct applicability and relevance of these findings.

A limitation of this study is that the plant material was collected from a single geographical origin (Urla, İzmir, Türkiye). Therefore, the findings should be interpreted with caution when extrapolating to *M. sylvestris* from other microecological regions or cultivation conditions, as chemotypic variation may influence infusion‐dependent extraction profiles.^3^, ^4^

Future research should therefore: (i) expand to multi‐regional and seasonal samples to assess chemotypic diversity; (ii) implement time‐resolved kinetic profiling (e.g., 0–30 min hot, 0–48 h cold) to model compound release dynamics; (iii) integrate gas chromatography‐olfactometry to identify true odor‐active volatiles; (iv) evaluate microbial stability and safety in cold‐brewed infusions under controlled hygiene conditions; and (v) validate health‐relevant effects through *in vitro* digestion models and cellular bioassays. Such efforts will bridge traditional knowledge with evidence‐based innovation, enabling the rational design of functional mallow teas that balance sensory pleasure, nutritional value, and product safety.

## CONFLICT OF INTEREST

The authors declare no conflict of interest.

## Data Availability

The data that support the findings of this study are available from the corresponding author upon reasonable request.
